# Structural Stability of *Burkholderia cenocepacia* Biofilms Is Reliant on eDNA Structure and Presence of a Bacterial Nucleic Acid Binding Protein

**DOI:** 10.1371/journal.pone.0067629

**Published:** 2013-06-14

**Authors:** Laura A. Novotny, Amal O. Amer, M. Elizabeth Brockson, Steven D. Goodman, Lauren O. Bakaletz

**Affiliations:** 1 Center for Microbial Pathogenesis, The Research Institute at Nationwide Children’s Hospital, and The Ohio State University College of Medicine, Columbus, Ohio, United States of America; 2 Department of Microbial Infection and Immunity, Center for Microbial Interface Biology and the Department of Internal Medicine, College of Medicine, and the Ohio State University, Columbus, Ohio, United States of America; Ghent University, Belgium

## Abstract

Cystic fibrosis (CF) is the most common lethal inherited genetic disorder affection Caucasians. Even with medical advances, CF is life-shortening with patients typically surviving only to age 38. Infection of the CF lung by *Burkholderia cenocepacia* presents exceptional challenges to medical management of these patients as clinically this microbe is resistant to virtually all antibiotics, is highly transmissible and infection of CF patients with this microbe renders them ineligible for lung transplant, often the last lifesaving option. Here we have targeted two abundant components of the *B. cenocepacia* biofilm for immune intervention: extracellular DNA and DNABII proteins, the latter of which are bacterial nucleic acid binding proteins. Treatment of *B. cenocepacia* biofilms with antiserum directed at one of these DNABII proteins (integration host factor or IHF) resulted in significant disruption of the biofilm. Moreover, when anti-IHF mediated destabilization of a *B. cenocepacia* biofilm was combined with exposure to traditional antibiotics, *B. cenocepacia* resident within the biofilm and thereby typically highly resistant to the action of antibiotics, were now rendered susceptible to killing. Pre-incubation of *B. cenocepacia* with anti-IHF serum prior to exposure to murine CF macrophages, which are normally unable to effectively degrade ingested *B. cenocepacia*, resulted in a statistically significant increase in killing of phagocytized *B. cenocepacia*. Collectively, these findings support further development of strategies that target DNABII proteins as a novel approach for treatment of CF patients, particularly those whose lungs are infected with *B. cenocepacia*.

## Introduction

Cystic Fibrosis (CF) is the most common inherited lethal disorder affecting Caucasians [1]. The greatest impact of this genetic disorder is the inability of CF patients to eradicate bacterial pathogens from their lungs. As a result, a chronic infectious state develops, which includes a vicious cycle of bacterial proliferation followed by retreat into an adhered extracellular biofilm state and/or invasion of local host cells. With each cycle of acute proliferation, an intense host response is elicited, and in an attempt to clear these infections, the over-exuberant inflammatory response causes damage to lung tissues, resulting in scarring that impairs pulmonary function and is often fatal. Even today, at least 90% of CF patients die from respiratory failure. Current treatment modalities rely heavily on antibiotic use, however, certain pathogens remain highly recalcitrant to antibiotic treatment, a phenotype that is significantly contributed to by their ability to reside within a biofilm. Once biofilm formation occurs and infection is established in the CF lung, eradication of bacteria is rare [2].

Whereas the major pathogen in adult CF patients is *Pseudomonas aeruginosa*, amongst the most deleterious CF-associated pathogens are members of the *Burkholderia cepacia complex* (*Bcc*), with perhaps the most virulent member being *Burkholderia cenocepacia*, a Gram-negative opportunistic pathogen. Of the 17 formally named species within the complex, *B* *. multivorans* and *B. cenocepacia* dominate in CF [3–6] accounting for approximately 85–97% of all *Burkholderia* infections. While CF patients infected with any *Bcc* species often have a poor prognosis, infection with *B. cenocepacia* is considered more serious and associated with both reduced survival and greater risk for development of fatal ‘cepacia syndrome’ [3–10]. *B. cenocepacia* is intrinsically resistant to polymyxins, aminoglycosides and most β-lactams, and can develop resistance to essentially all classes of antimicrobial drugs [11–13].

*B. cenocepacia* gained notoriety as a pathogen in CF because it is difficult to identify and treat, and also due to its ability to readily spread between individuals with CF. Even outside CF, highly transmissible epidemic strains of *B. cenocepacia* have been shown to contaminate healthcare settings and spread nosocomially [14]. It is also likely that multi-drug resistant *B. cenocepacia* can transfer mechanisms of resistance to other microbes present within the human airway, and particularly those co-colonizing the CF lung. This assumption is supported by the observation that 25-45% of adult CF patients are chronically infected with multiple multi-drug resistant bacteria [15].

The pathogenesis of CF airway disease is multifactorial and includes defective antimicrobial activity in airway secretions, altered mucociliary clearance, abnormal sub-mucosal gland function and overproduction of reactive oxygen species (ROS). Chronic inflammation is most central to CF pathogenesis as a consequence of pulmonary infections and leads to lung damage resulting in 85% of deaths. Further contributing to enhanced lung pathology observed in CF patients is an overabundance of cytokine-secreting alveolar macrophages. Pro-inflammatory mediators such as IL-1β, IL-8, TNF-α and anti-inflammatory IL-10 are detected in CF patients, even in young patients in the absence of culture-positive infection. Moreover, whereas alveolar macrophages typically engulf microbes that gain access to the lungs, enclose them into vacuoles which fuse with lysosomes where the contents are degraded and eliminated via a process called autophagy, CF macrophages are defective in this regard. This inherent weak autophagy [16–18] is further augmented by the ability of *B. cenocepacia* to downregulate essential autophagy molecules [19].

Infection of a CF patient with *B. cenocepacia* is considered a virtual death sentence as these patients are extremely difficult to treat and are also ineligible for lung transplant, often their last lifesaving resort. Thereby, the need to develop novel, highly effective approaches to eradicate *B. cenocepacia* from the lungs of CF patients, as well as from medical environments, cannot be underestimated. We have recently identified a protein target for the development of such a novel approach. This protein, a member of the DNABII family of DNA binding proteins, is essential for biofilm formation and stability by multiple human pathogens [20–22] due to its contribution to the structural lattice of extracellular DNA (eDNA) within these bacterial communities. The DNABII family is a member of a class of proteins referred to as nucleoid associated proteins (NAPs), bacterial proteins that, in part, shape the intracellular bacterial nucleoid [23]. In addition, this family is ubiquitous, expressed by virtually all eubacteria. All characterized family members to date function as either a homodimer or heterodimer of subunits. The family is divided into two types, HU (histone-like protein) and IHF (integration host factor) with *B. cenocepacia* capable of expressing both (strain J2315 genes: BCAL3530, *hupA*; BCAL1585, *hupB*; BCAL1487, *ihfA* and BCAL2949, *ihfb*). The primary distinction between these family members is that HU binds DNA in a sequence independent manner, while IHF binds a consensus sequence [WATCAANNNNTTR where W is A or T and R is a purine) conserved across *genera* [24]]. All DNABII proteins bind to and bend DNA considerably e.g. *E. coli* IHF can bend DNA into a virtual U-turn [25]. In addition, all family members have a preference for pre-bent or curved DNA structures e.g. Holliday junctions, a cruciform-like structure central to DNA recombination. In fact, DNABII proteins function as accessory factors facilitating all intracellular DNA functions, including gene expression, recombination, repair and replication [24].

In the past 20 years, multiple investigators discovered that these proteins also exist extracellularly [26–29]. To date, three extracellular functions have been described. First, streptococcal HU is shown to elicit a powerful inflammatory innate immune response by inducing the release of TNFα and interleukin-1 [30]. Interestingly, *B. cenocepacia* induces peripheral damage by exacerbating IL-1β production through the host receptor Pyrin by an as yet unknown mechanism [31,32]. In this regard, an extracellular role of DNABII family members has yet to be tested for *B. cenocepacia*. Second, extracellular DNABII proteins bind laminin and are thought to elicit direct contact to host cells [27]. Third, DNABII proteins are known to stabilize the structural integrity of eDNA within the extra-cellular polymeric matrix or substance (or EPS) of the biofilm of multiple pathogens [20]. Antisera directed against these proteins are sufficient to destabilize biofilms, resulting in exposure/release of the resident bacteria, thus sensitizing them to the action of both antimicrobials and effectors of the immune system [20]. Recently, it’s also been shown that both IHF subunits are required for efficient colonization of the urinary bladder by uropathogenic *E. coli* and further, that both IHF subunits influence the community architecture of intracellular bacterial communities [21]. Considering the abundance of eDNA within a *B. cenocepacia* induced biofilm, we first hypothesized that there might be a role for the DNABII family of proteins in stabilization of these biofilms as well and thus, this family of proteins might serve as a target for therapeutic intervention. Moreover, we also wondered if the presence of extracellular DNABII proteins, perhaps in association with bacterial cells, might influence the interaction of *B. cenocepacia* with host cells and particularly with its primary reservoir, the macrophage.

Here we show that an immuno-therapeutic approach that targets the DNABII proteins, and particularly when coupled with existing conventional therapeutics was highly effective at both debulking biofilms formed by *B. cenocepacia* as well as rendering the bacteria now released from the biofilm EPS susceptible to the action of antibiotics. These findings suggested that with further development, this approach could perhaps be highly effective to diminish, and even perhaps eradicate reservoirs of *B. cenocepacia* from the lungs of CF patients. Moreover, this strategy showed promise for preventing *recurrence* of disease by inhibiting the ability of *B. cenocepacia* to multiply within macrophages isolated from CF mice, an important animal model of CF in humans.

## Materials and Methods

### Ethics statement

Prior to collection of sputa from pediatric patients at Nationwide Children’s Hospital, written informed consent and authorization was obtained from the parent(s) or a legally authorized representative. This procedure was in concordance with all institutional and federal guidelines and was performed according to a protocol approved by the Institutional Review Board at Nationwide Children’s Hospital, Columbus, Ohio.

### Biofilm formation *in vitro*

*B. cenocepacia* strains (including strains K56-2, DFA2 and JRL2) were inoculated from a frozen stock into LB broth (BBL) and grown overnight at 37°C with shaking at 200 rpm. Cultures were then diluted 1:100 in fresh broth, the optical density read at 600 nm and adjusted to a final concentration of 10^6^ CFU *B. cenocepacia*/ ml medium. The bacteria were further diluted 1:2500 in LB broth and 200 µl of the bacterial suspension was added to each well of a LabTek II 8-well chambered coverglass (LabTek). Slides were incubated static for 16 hrs at 37°C in a humidified atmosphere at which time medium was aspirated and replaced with fresh LB broth. After an additional 8 hrs (24 hrs total incubation time), biofilms were either stained or treated with antiserum or antibiotic. Assays were performed a minimum of three times.

### Distribution of IHF and DNA within a *B. cenocepacia* biofilm

To examine the relative distribution of IHF within biofilms formed by *B. cenocepacia* strains, unfixed 24 hr biofilms were incubated with naive rabbit serum or rabbit anti-*E. coli* IHF [20] and revealed with goat anti-rabbit IgG conjugated to AlexaFluor 594 (Invitrogen). DNA within the biofilms was stained with anti-dsDNA antibody (Abcam, Inc.) and revealed with goat anti-mouse IgG conjugated to AlexaFluor 647 (Molecular Probes). Intact bacterial cells were stained with the membrane stain FilmTracer™ FM^®^ 1-43 Green Biofilm Stain (Molecular Probes) according to manufacturer’s instructions. Images were collected with a Zeiss 510 meta-laser scanning confocal microscope (Carl Zeiss) using a 63X objective. 3-D images were reconstructed with AxioVision Rel. 4.8 (Carl Zeiss).

To determine the relative abundance of eDNA in a 24 hr biofilm formed by *B. cenocepacia*, the DNA to bacterium ratio was determined using Zeiss image acquisition software wherein the relative fluorescent intensity of anti-dsDNA labeling to FilmTracer™ was compared. Biofilms formed by nontypeable *Haemophilus influenzae* were also established [20,33] and similarly stained.

### Distribution of IHF within human sputa

To visualize IHF labeling with human sputa, sputum samples were collected from 3 pediatric patients with CF that were also culture positive for *B. cenocepacia* under an approved IRB protocol. Sputa were embedded in OCT compound (Fisher Scientific) and snap frozen in the vapor phase of liquid nitrogen. Ten micron serial sections were cut and adhered to glass slides. Slides were air-dried, fixed in cold acetone, equilibrated in buffer containing 0.05M Tris-HCl, 0.15M NaCl and 0.05% Tween 20 (pH 7.4) then blocked with image-iT FX signal enhancer (Molecular Probes) and Background Sniper (BioCare Medical). Sections were incubated with polyclonal anti-IHF or naive rabbit serum, and revealed with goat anti-rabbit IgG-Alexafluor 594 (Invitrogen). DNA was counterstained with Prolong Gold anti-fade reagent with DAPI (Molecular Probes). Images were viewed as described prior.

### Resolution of established *B. cenocepacia* biofilms

To assess the ability of antiserum to resolve a biofilm formed *in vitro*, 24 hr biofilms were first established using strain K56-2 as described, then exposed to either naive rabbit serum or rabbit anti-*E. coli* IHF diluted 1:50 in LB broth, or LB broth only, for 16 hrs. Biofilms were then stained with LIVE/DEAD^®^ *Bac*Light™ Bacterial Viability kit for microscopy (Molecular Probes) as previously described [33], then fixed with a solution of 16% paraformaldehyde- 2.5% glutaraldehyde- 4% acetic acid in 0.2M phosphate buffer, pH 7.4, prior to immediate imaging by confocal microscopy as described prior. Quantitation of average thickness and mean biomass was determined via COMSTAT2 analysis [34]. As an additional measure to confirm that application of naive and immune rabbit serum did not induce cell death, we collected both planktonic and biofilm adherent *B. cenocepacia* 16 hrs after treatment, stained the unfixed cells with LIVE/DEAD^®^ *Bac*Light™ Bacterial Viability and Counting kit for flow cytometry (Molecular Probes) and distinguished between live and dead bacteria using a C6 calibrated flow cytometer (Accuri). Assays were performed a minimum of three times.

### Enrichment of IgG from whole serum

To confirm that biofilm resolution was mediated by antibody and not other serum components, we purified IgG from rabbit anti-IHF serum with HiTrap Protein G HP columns (GE Healthcare) according to manufacturer’s instructions and collected both the eluent that passed through the column after application of serum and the IgG-enriched fraction. To demonstrate that anti-IHF and IgG-enriched anti-IHF recognized purified IHF in addition to native IHF expressed by *B. cenocepacia* strain K56-2, we performed SDS-PAGE and Western blotting. Briefly, 0.5 µg purified IHF ( [25]; the gift of Howard Nash, NIH) and 5 µg *B. cenocepacia* whole cell lysate were separated on a 4-15% Mini-PROTEAN TGX gel (BioRad) in Tris/Glycine/SDS buffer (BioRad) then transferred on to nitrocellulose membranes (Invitrogen). Membranes were blocked overnight at 4°C in 3% skim milk in Tris-buffered saline with 0.5% Tween 20 (Fisher Scientific) prior to incubation with rabbit anti-IHF or IgG-enriched anti-IHF followed by goat anti-rabbit IgG-HRP (Invitrogen). Blots were developed with CN/DAB substrate kit (Pierce) and images captured with BioRad GS800 densitometer.

### Synergistic effect of anti-IHF and antibiotics in biofilm resolution

Examination of the synergistic effect of antibiotics combined with antiserum directed against IHF when applied to a biofilm formed by *B. cenocepacia* strain K56-2 required determination of the minimum inhibitory concentration (MIC) of several antibiotics for planktonic cultures of *B. cenocepacia* strain K56-2. To do so, 100 CFU *B. cenocepacia* were inoculated into LB broth containing two-fold serial dilutions of the following antibiotics: ceftazidime, ciprofloxacin, imipenem, meropenem, minocycline, sulfamethoxazole-trimethoprim or tobramycin (Sigma). Cultures were incubated static for 24 hrs, and turbidity of the culture assessed. The MIC for planktonic *B. cenocepacia* was identified as the concentration of antibiotic at which no bacterial growth was observed. With this information, established *B. cenocepacia* biofilms were treated with a 1:50 dilution of anti-IHF, antibiotic at the determined MIC, anti-IHF plus antibiotic or medium alone for 16 hrs prior to viability stain with LIVE/DEAD^®^ *Bac*Light™ Bacterial Viability kit and visualization by confocal microscopy as previously described. The following concentration of antibiotics were used: ceftazidime- 16 µg/ml, ciprofloxacin- 2 µg/ml, imipenem- 32 µg/ml, meropenem- 16 µg/ml, minocycline- 4 µg/ml, sulfamethoxazole-trimethoprim- 16 µg/ml and tobramycin- 512 µg/ml. Quantitation of average thickness and mean biomass was determined via COMSTAT2 analysis. Assays were performed a minimum of three times.

### Treatment of established biofilms with Pulmozyme^®^

To examine the outcome of treatment of established *B. cenocepacia* strain K56-2 biofilms with Pulmozyme^®^ (dornase alpha; Genentech, Inc.), established biofilms were treated with a 1:50 dilution of anti-IHF, 0.25 mg of Pulmozyme^®^, anti-IHF plus Pulmozyme^®^ or medium alone for 16 hrs prior to viability stain and visualization by confocal microscopy. Quantitation of average thickness and mean biomass was determined via COMSTAT2 analysis. Assays were performed a minimum of three times.

### Impact of anti-IHF antibody on *B. cenocepacia* survival within macrophages

*B. cenocepacia* strain MHK1 [35] which has a mutation in an antibiotic efflux pump that confers gentamicin sensitivity but does not alter trafficking of the mutant in macrophages was used for this assay. Strain MHK1 was cultured as described above then treated with naive serum or anti-IHF serum at a concentration of 1:1000 for 15 minutes. Treated *B. cenocepacia* were then added to murine bone marrow -derived macrophages for 1 hr. To kill extracellular bacteria, Iscove’s media (GIBCO) containing 10% heat-inactivated FBS (GIBCO) and 50 µg gentamicin/ml (GIBCO) was added for 30 min. Macrophages were lysed at 2, 4 and 6 hrs post-infection and lysates were plated to determine CFU *B. cenocepacia*/ ml. Assays were performed a minimum of three times.

### DNase footprinting experiments

The 386 bp of upstream DNA from *BCAL0339* and *BCAL0340* was amplified by the PCR reaction. The isotopically end labeled (^32^P) amplicon was used in a binding reaction with crystallographically pure *E. coli* IHF ([25]; the gift of Howard Nash, NIH) and subsequently in DNase I footprinting assays [36].

Statistical methods. To determine significant differences in average biofilm thickness and biomass, and in macrophage association assays, two sample paired T-tests were performed using GraphPad Prism software, version 6.00. 

## Results

### Evidence for the presence of abundant extracellular DNA (eDNA) in biofilms formed by *B. cenocepacia* in vitro

**Figure 1:**
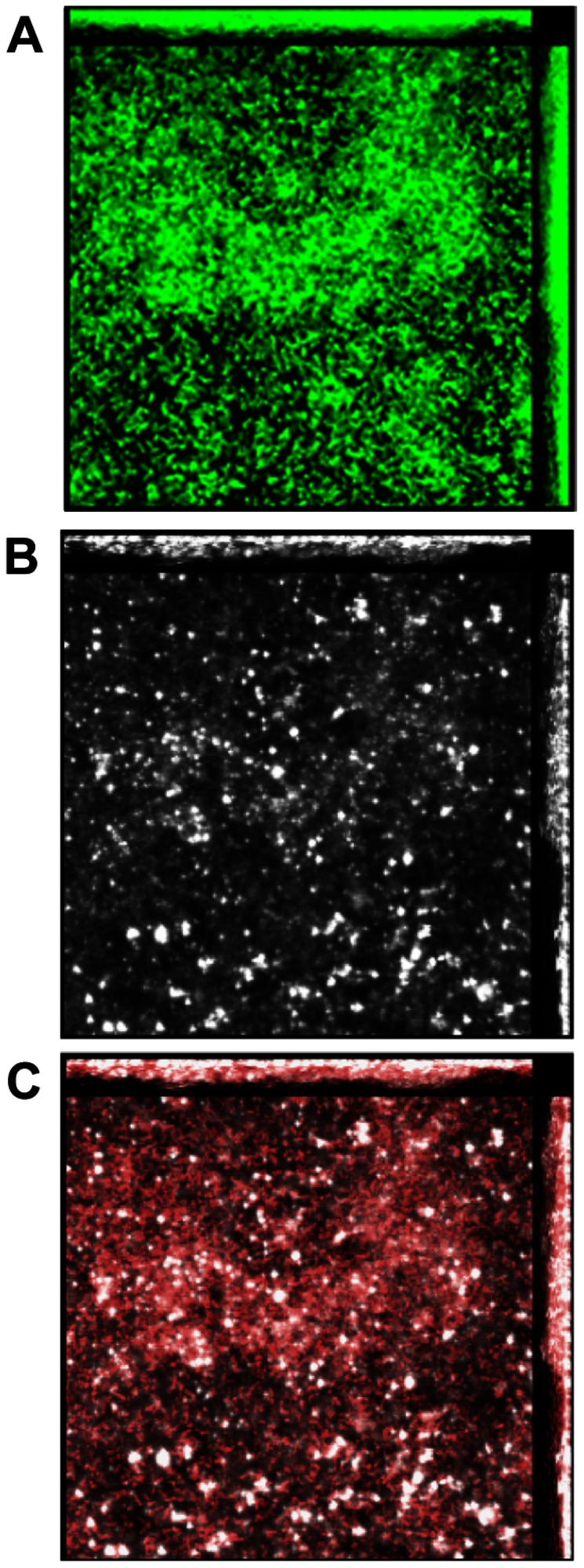
Presence of both eDNA and IHF within a biofilm formed by*B. cenocepacia*. **Panel A** – Unfixed twenty-four hr biofilm formed by *B. cenocepacia* stained with FilmTracer FM 1-43. **Panel B** – unfixed 24 hr biofilm formed by *B. cenocepacia* and immunolabeled with monoclonal antiserum directed against dsDNA (white color in image). **Panel C** – unfixed 24 hr. biofilm formed by *B. cenocepacia* labeled with both antiserum directed against dsDNA and rabbit anti-IHF serum to show the presence of IHF (red color) as well as dsDNA (white color) within the biofilm. Note robust biofilm formed by *B. cenocepacia* in Panel A. The presence of eDNA that is particularly dense at the bottom of the biofilm can be seen in Panel B. The 24-hr biofilm formed by *B. cenocepacia* is also rich in IHF as shown by the abundance of red color in Panel C. All images captured with a 63X objective.

To first characterize the basic structure of *B. cenocepacia* biofilms when formed *in vitro*, we grew *B. cenocepacia* strain K56-2 statically in a chamber slide for 24 hrs before labeling with FilmTracer FM 1-43. As shown in Figure 1A, B *. cenocepacia* formed a robust biofilm of approx. 26 µm height with characteristic towers. To now determine whether *B. cenocepacia* incorporated DNA into its biofilm matrix, we labeled the unfixed biofilm with a monoclonal antibody to detect the presence of dsDNA (white). The biofilm formed contained an abundant amount of eDNA that was particularly dense at the base of the biofilm (Figure 1B), suggesting that this matrix component might be serving an essential role in bacterial adherence and anchoring during early stages of biofilm development. Interestingly, while visually apparent, further COMSTAT analysis of both a *B. cenocepacia* biofilm and one formed by nontypeable *Haemophilus influenzae* (NTHI) (data not shown) then labeled in an identical manner and using separate laser channels (to detect bacteria as green and DNA as a white signal) confirmed in a more objective manner that *B. cenocepacia* incorporated approximately 30% more eDNA per bacterial cell into its biofilm than does this additional important human airway pathogen [37].

### Demonstration of a DNABII protein within biofilms generated by *B. cenocepacia*

To determine whether biofilms formed by *B. cenocepacia* strain K56-2 contained a member of the DNABII family of proteins, we incubated a biofilm formed *in vitro* by *B. cenocepacia* strain K56-2 with rabbit anti-IHF antibody [antiserum against *E. coli* IHF that cross-reacts with multiple DNABII family members [20]] and observed labeling (red) throughout the biofilm (Figure 1C).

**Figure 2:**
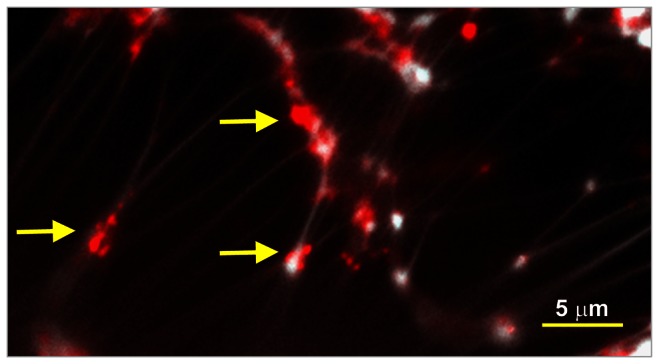
Labeling of IHF and eDNA within sputum collected from a CF patient culture-positive for *B. cenocepacia*. Immunolabeled light micrograph demonstrates heavy labeling of both eDNA (white strands) and the DNABII protein IHF (punctate red labeling; see yellow arrows) within a sputum sample recovered from a CF patient infected with *B. cenocepacia*. As previously shown with nontypeable *Haemophilus influenzae*-formed biofilms, in those formed by *B. cenocepacia*, junctions where strands of bacterial eDNA cross label strongly for the presence of IHF, suggesting their role in maintaining the structural scaffolding of these biofilms. Scale bar equals 5 µm.

To now assess whether our observations made *in vitro* were relevant to the clinical condition of CF patients, as well as to determine whether, as we had seen with NTHI [20], IHF was positioned at the vertices of crossed strands of eDNA present within the biofilm formed by *B. cenocepacia*, we obtained sputum samples from CF patients known to be infected with *B. cenocepacia*. Samples were snap frozen and immunolabeled using the same anti-IHF antibody. We observed heavy labeling of 3/3 (100%) of sputum samples recovered to date. Notably, we observed specific labeling of virtually 100% of the vertices formed by overlapping dsDNA strands present within these samples (Figure 2). Given the large amount of eDNA present within the EPS matrix of a *B. cenocepacia*-produced biofilm, and the fact that IHF appeared to be critically positioned within this matrix, we hypothesized that targeting this protein for intervention might result in structural collapse of the biofilm. If this collapse occurred, we further reasoned that this might greatly facilitate access of immune effectors, antibiotics or other therapeutic agents to bacteria formerly protected by the EPS, thus promoting their eradication.

### Demonstration of the ability of anti-IHF to disrupt a biofilm formed by *B. cenocepacia* in vitro 

**Figure 3:**
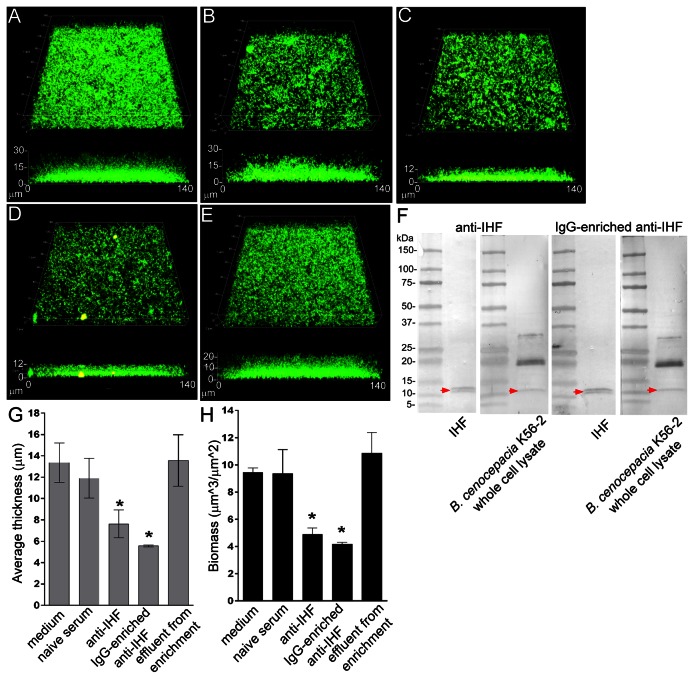
Disruption of pre-formed *B. cenocepacia* biofilms by incubation with antiserum directed against IHF. *B. cenocepacia* biofilms after 24 hr. growth in a chamber slide, then treated for 16 hrs with: **Panel A**-sterile medium. **Panel B** – naive rabbit serum. **Panel C** –rabbit antiserum directed against isolated IHF. **Panel D –**IgG enriched anti-IHF. **Panel E** –serum effluent from enrichment column. **Panel F** – Western blot showing recognition of antibody directed against IHF as well as IgG-enriched anti-IHF to purified IHF and IHF within *B. cenocepacia* whole cell lysate. Red arrows indicate recognition of the monomer form of IHF in each blot. Moreover, each serum fraction recognized the di- and tri-meric forms of IHF within whole cell lysates of *B. cenocepacia* strain K56-2. **Panel G** – plot of changes in average biofilm thickness ± SEM following each of the indicated treatments. **Panel H** – plot of changes in biofilm biomass ± SEM following each of the indicated treatments. Note that statistically significant disruption of pre-formed *B. cenocepacia* biofilms is mediated only by rabbit anti-IHF serum as well as a fraction of that serum that was enriched for IgG when compared to treatment with sterile medium, naive rabbit serum or serum effluent from IgG enrichment column. Asterisks indicate statistical significance (*p* < 0.05) compared to sterile medium, naive serum and enrichment column effluent.

To test one of these hypotheses, we treated 24 hr *B. cenocepacia* strain K56-2 biofilms with either medium (Figure 3A), naive rabbit serum (diluted 1:50) (Figure 3B) or rabbit anti-IHF serum (at an arbitrarily selected dilution of 1:50) (Figure 3C). Following 24 hrs of incubation, we re-analyzed these biofilms via COMSTAT software and found that treatment with naive rabbit serum induced a small and statistically non-significant change in biofilm thickness but had no effect on mean biomass over that seen when sterile medium was used (Figure 3G& 3H). This effect is attributed to non-specific antibodies within whole rabbit serum that cross react with outer membrane proteins of multiple Gram negative bacteria. Conversely however, treatment with antiserum directed against IHF resulted in: 44% reduction in thickness, 56% reduction in biomass, and 52% reduction in height compared to naive serum. These latter effects were statistically significant compared to use of either sterile medium or naive serum (Figure 3G& 3H). To demonstrate that treatment with antiserum directed against IHF did not kill resident bacteria thus accounting for the observed disruption of the biofilms, after treatment we recovered both the planktonic sub-population of bacteria contained within the culture medium as well as that bacterial sub-population that remained within the biofilm and subjected both preparations to analysis by flow cytometry to determine relative proportion of live to dead bacteria. Regardless of treatment used, we found that ≥80% of bacteria within the planktonic sub-population and ≥93% of those that has remained resident within the biofilm were viable. This result suggested that treatment with anti-IHF mediated the release of viable bacteria into the planktonic phase.

To demonstrate that the biofilm disruption activity observed upon treatment with rabbit anti-IHF serum was primarily due to IgG antibodies directed against IHF in this polyclonal but hyperimmune serum, we enriched this serum for IgG as described in Methods then assayed both the fraction enriched for IgG (Figure 3D) as well as the serum fraction that flowed through the column (Figure 3E) for relative ability to disrupt a pre-formed *B. cenocepacia* biofilm. As can be seen in Figure **3D& 3E**, as well as plotted in **3G&3H**, the IgG enriched antiserum preparation retained the activity of the whole rabbit anti-IHF; however there was no ability of the effluent from the enrichment column to similarly disrupt the biofilm. Western blots were performed to demonstrate that antibodies contained within both whole anti-IHF serum as well as the fraction enriched for IgG recognized purified IHF protein as well as the mono-, di- and tri-meric forms of this protein within whole cell lysates of *B. cenocepacia* strain K56-2 (Figure 3F); the DNABII family member, PG0121 (HUβ from *Porphyromonas gingivalis*), also shows the capacity to maintain multimers during SDS PAGE (S. Goodman, personal communication).

### Demonstration of synergistic interaction between anti-IHF and traditional antibiotics

**Figure 4:**
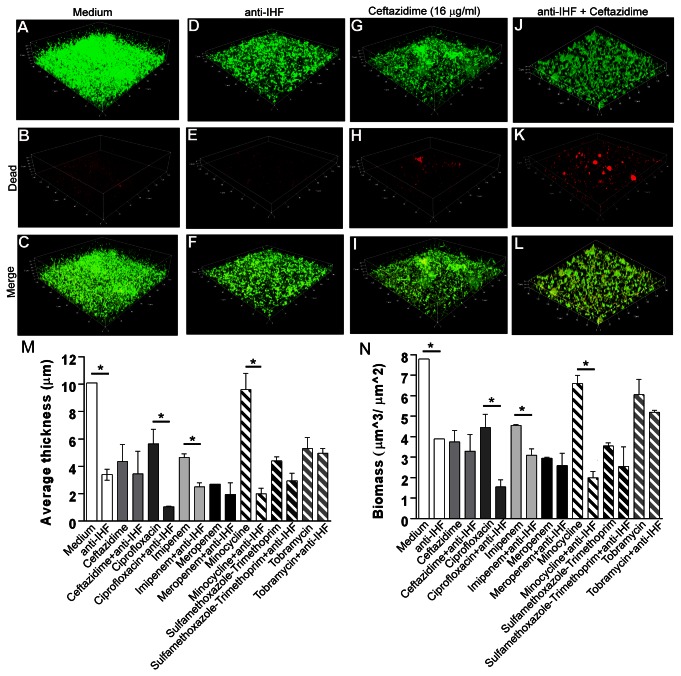
Demonstration of the synergistic behavior of antibodies directed against IHF in combination with antibiotics. **Panels A-C** - An untreated *B. cenocepacia* biofilm. **Panels D–F** – a *B. cenocepacia* biofilm after treatment with a 1:50 dilution of anti-IHF. **Panels G-I** – a *B. cenocepacia* biofilm after treatment with the MIC of ceftazidime (16 µg/ml). **Panels J-L** – a *B. cenocepacia* biofilm after treatment with a combination of anti-IHF plus ceftazidime at the noted MIC. Note marked reduction of biofilm height and notably increased killing of *B. cenocepacia* when treated with both anti-IHF and ceftazidime compared to treatment with antibiotic alone (indicated by red/orange color in second row of images; compare Panels H and K). Panel M – average thickness of biofilms after treatment with antibiotic or antibiotic plus anti-IHF ± SEM. Panel N – Biofilm biomass following incubation with antibiotic or antibiotic plus anti-IHF ± SEM. Asterisks indicate statistical significance between designated pairs (*p*< 0.05). Note significant reduction in both average biofilm thickness and biomass as mediated by a combination of anti-IHF serum plus ceftazidime, ciprofloxacin, imipenem and minocycline.

To ascertain whether anti-IHF mediated biofilm disruption might now render resident bacteria more susceptible to treatment with other existing potential therapeutic agents, we exposed pre-formed *B. cenocepacia* biofilms to each of seven antibiotics (using the determined MIC for each against planktonically grown *B. cenocepacia* strain K56-2 as described in Experimental Procedures) either alone or in combination with anti-IHF serum (at a 1:50 dilution). As shown in Figure **4** [using incubation with ceftazidime (at 16 µg/ml) as an example], whereas treatment with anti-IHF (Figure 4D) induced a reduction in biofilm height, thickness and biomass over that of an untreated biofilm (Figure 4A), neither of these treatments induced much bacterial cell death (Figure 4B& E). Further, antibiotic treatment alone had little observable effect on the *B. cenocepacia* induced biofilm (Figure 4G), and while bacterial cell death had increased over that observed following treatment with either sterile medium or anti-IHF alone, this effect was nonetheless minimal (Figure 4H). When used together however, there was a 43% reduction in height (Figure 4J) and an obvious and notable increase in bacterial cell death (as indicated by red/orange color) (Figure 4K) over that observed when antibiotics were used alone (compare Figure 4H& K), suggesting a synergistic interaction between anti-IHF and ceftazidime. A significant reduction in average biofilm height (Figure 4M) and biomass (Figure 4N) between treatment with anti-IHF plus antibiotic versus antibiotic alone was similarly obtained following treatment of *B. cenocepacia* biofilms with ciprofloxacin plus anti-IHF, imipenem plus anti-IHF and minocycline plus anti-IHF (*p*< 0.05).

### Demonstration that use of Pulmozyme (DNase) may be contraindicated in CF patients that are infected with *B. cenocepacia*

**Figure 5:**
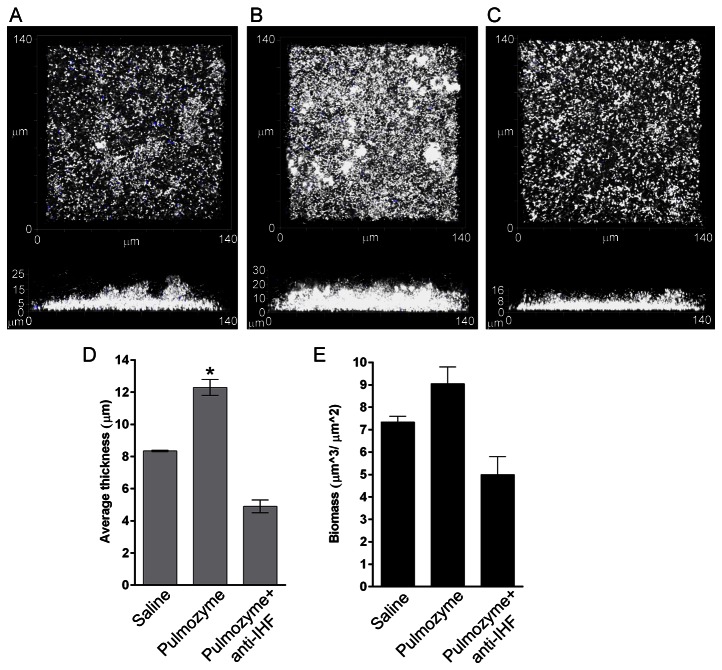
Induction of a more robust*B. cenocepacia* biofilm following exposure to Pulmozyme (DNase). **Panel A** – Treatment of a 24 hr *B. cenocepacia* biofilm with saline diluent alone. **Panel B** - Treatment of a 24 hr. *B. cenocepacia* biofilm with Pulmozyme (DNase) induced the formation of a markedly denser and thicker biofilm than that treated with saline diluent alone (compare panels A and B). **Panel C** – treatment of a 24 hr *B. cenocepacia* biofilm with both Pulmozyme and anti-IHF. Biofilms were stained for viability and pseudocolored white (live cells) and blue (dead cells) and demonstrate minimal bacterial death upon any treatment. Mean relative biofilm thickness ± SD and biomass ± SEM are depicted graphically in **Panels E & F**. Asterisk indicates significantly thicker biofilm after exposure to Pulmozyme compared to treatment with saline diluent (*p*< 0.05).

Treatment with DNase is used as a standard of care for patients with CF, and given that *B. cenocepacia* incorporates an abundance of eDNA into its biofilm, we hypothesized that use of anti-IHF in combination with DNase would also potentially have a synergistic effect in terms of debulking and eradicating bacteria within a *B. cenocepacia*-induced biofilm *in vitro* as we previously showed occurs with NTHI [20]. However, this was not the case. In fact, in multiple repeated assays, exposure of a *B. cenocepacia* biofilm to DNase induced a significantly more robust biofilm in terms of average thickness (Figure 5B& D) than one treated with diluent alone (Figure 5A). These DNase-treated biofilms were only slightly increased in maximum height (compare 32 µm to 28 µm), however the mean increase in surface to volume ratio was *12%*; the mean increase in biomass was *174%* and when compared for average thickness, and the biofilm formed by *B. cenocepacia* that had been treated with DNase was *204%* thicker than that formed by *B. cenocepacia* that had been treated with diluent alone. Whereas antiserum directed against IHF was still able to effectively reduce these enhanced biofilms (Figure 5**C,D**& **E**), given the unexpected results obtained with DNase alone, pursuit of this line of investigation for potential synergistic use became counterintuitive. Importantly, this result has significant potential clinical implications for CF patients infected with *B. cenocepacia* as repeated use of DNase may actually *exacerbate* their disease and contribute to the very poor clinical outcome that exists for these patients.

### Demonstration that incubation of *B. cenocepacia* with antibodies directed at a DNABII protein but not naive serum results in reduced bacterial recovery from macrophages

**Figure 6:**
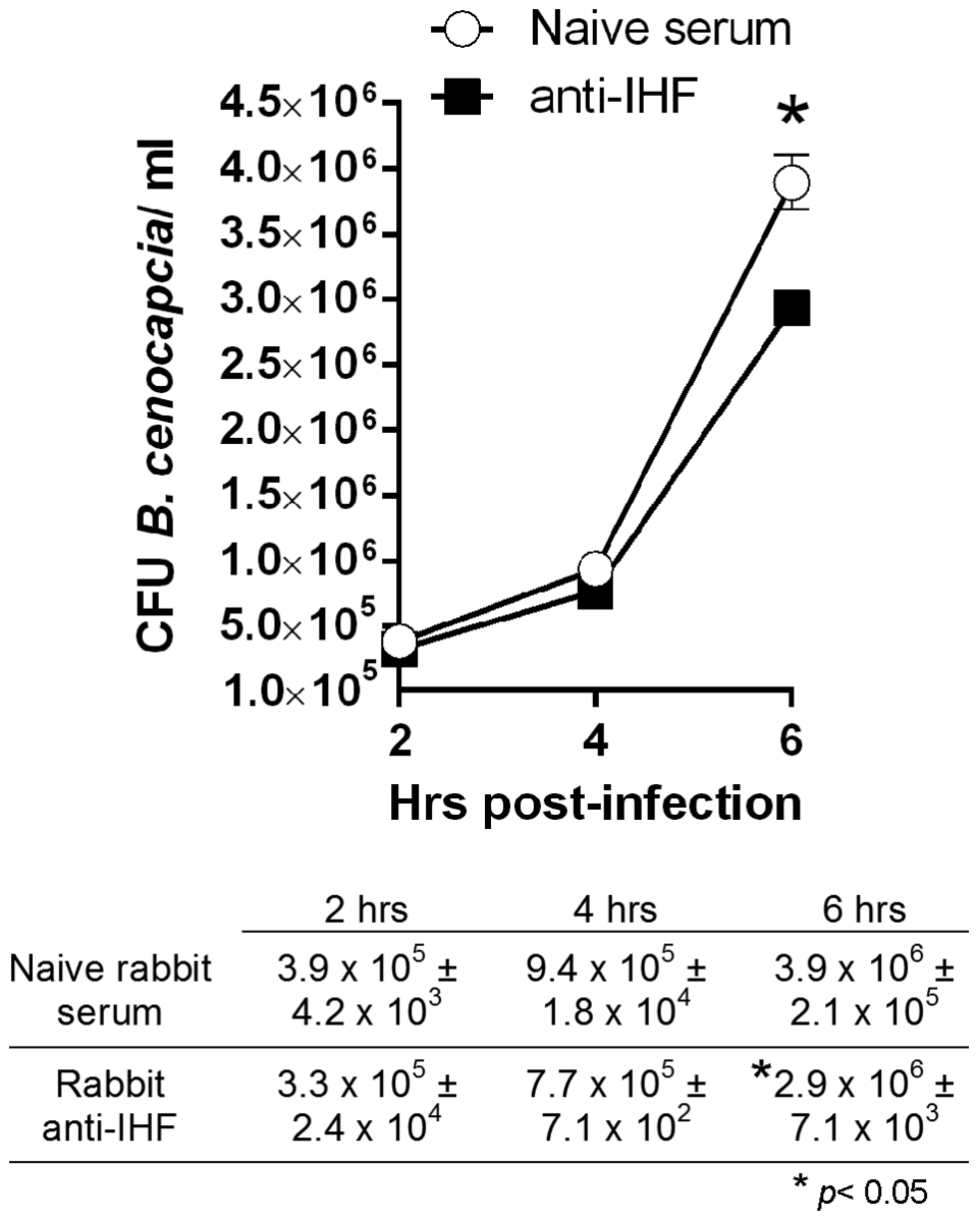
Pre-treatment of*B. cenocepacia* with anti-IHF induced significantly reduced survival in murine CF macrophages. Significantly fewer *B*. *cenocepacia* were detected 6 hrs after pre-treatment of the bacteria with anti-IHF compared to naive serum (* *p* < 0.05). Bacterial CFU/ml for each time point ± SD are also shown.

*B. cenocepacia* can persist and replicate within murine and human CF macrophages. However, WT murine macrophages or macrophages derived from n o n-CF patients restrict *B. cenocepacia* infection by delivery of the organism to the lysosome for degradation. Given that it has recently been demonstrated that IHF associated with uropathogenic *E. coli* influences the ability of this bacterium to efficiently colonize the bladder [21], we wondered if incubation of *B. cenocepacia* with antiserum directed against IHF might influence its interaction with macrophages, given the importance of this host cell in clearance of bacteria from the CF lung. Using a variant of strain K56-2 where gentamycin sensitivity was introduced (strain MHK1); we determined the number of *B. cenocepacia* that multiplied intracellularly (after killing extracellular bacteria via gentamycin treatment). Whereas the number of macrophage-associated *B. cenocepacia* at 2 hours post infection was not significantly different between those pre-treated with naive serum *versus* those pre-treated with anti-IHF serum (at a 1:1000 dilution) which suggested equivalent uptake by macrophages at this time point, by 6 hrs post infection *B. cenocepacia* pre-treated with anti-IHF serum were significantly impeded for growth within CF macrophages (*p* < 0.05) (Figure 6). Thus, pre-treatment of *B. cenocepacia* with anti-IHF promoted their clearance by CF macrophages; however the mechanism which underlies this observation remains to be determined and is the subject of ongoing investigation.

### Demonstration of the association of T3SS or T6SS with incorporation of DNABII protein(s) into the *B. cenocepacia* biofilm matrix

**Figure 7:**
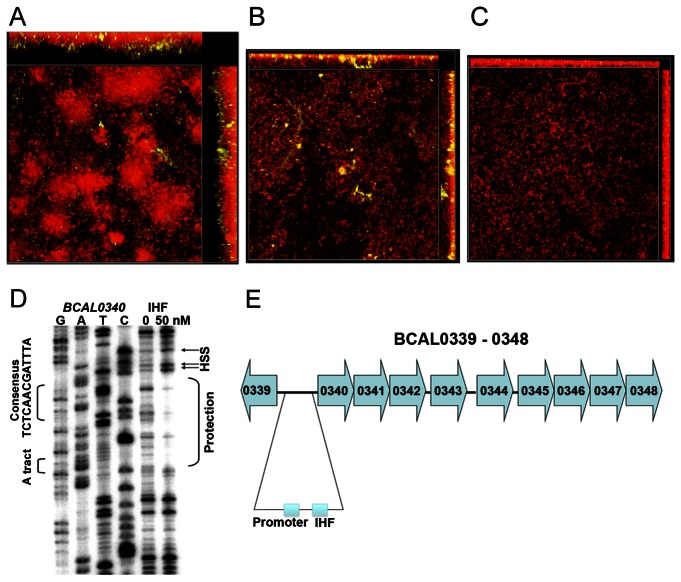
Expression of a robust *B. cenocepacia* biofilm that incorporates IHF was dependent upon an active T6SS. **Panel A** - Biofilm formed by the parental isolate (*B. cenocepacia* strain K56-2). **Panel B** - biofilm formed by the type III secretion system mutant (strain JRL2) stained with propidium iodide. **Panel C** – biofilm formed by the type VI secretion system mutant (strain DFA2) stained with propidium iodide. All biofilms were labeled for the presence of a DNABII protein (IHF) – see yellow color in images. Whereas biofilms formed by either secretion system mutant were notably less robust than that formed by the parental isolate, note marked reduction in yellow labeling of biofilm formed by the T6SS mutant (see Panel C), which suggested that the T6SS mutant was compromised in its ability to incorporate IHF into biofilms formed under these conditions. **Panel** D-IHF bound DNase footprint of the intergenic space (386 bp) between BCAL0339 and BCAL0340, part of the T6SS gene cluster for *B. cenocepacia*. The IHF footprint covers the region from 25 bp to 52 bp upstream of the BACL0340 start codon while a putative promoter was found 75 bp to 104 bp upstream of the start codon. HSS: hyper-sensitive site indicative of DNA bending. This observation suggested that IHF might self-regulate its own release as well as perhaps that of eDNA that is incorporated into the biofilm matrix.

To begin to attempt to elucidate the molecular mechanism(s) by which *B. cenocepacia* incorporates both eDNA and DNABII protein(s) into their biofilms, we incubated biofilms formed in 24 hrs by either *B. cenocepacia* strain K56-2 (parental isolate), *B. cenocepacia* strain JRL2 (Δ*bcsV*; type III secretion system mutant – T3SS) or *B. cenocepacia* strain DFA2 (Δ*BcsK*; type VI secretion system mutant – T6SS) with anti-IHF antibody [38]. In the biofilm formed by the parental isolate, positive labeling was distributed through the biofilm (Figure **7** **, Panel A**) as described earlier. In the biofilm formed by the T3SS mutant, whereas the biofilm itself was overall less robust than that formed by the parental isolate (biomass 4.5 µm^3^/µm^2^ compared to 18 µm^3^/µm^2^ for the parental isolate; average thickness 5.5 µm compared to 25.2 µm for the parental isolate), labeling via anti-IHF serum was again present throughout the biofilm, however as observed with the parental isolate, labeling appeared to be much stronger at the base of the biofilm (Figure **7** **, Panel B**). Conversely, however, there was very little labeling of the biofilm formed by the T6SS mutant (Figure **7** **, Panel C**), which was also significantly less robust than that formed by the parental isolate (biomass 2.9 µm^3^/µm^2^; thickness 3.9 µm). This latter observation was not unexpected as the T6SS of *B. cenocepacia* is known to be associated with biofilm formation [39]. Collectively however, these data suggested that export of DNABII proteins (and possibly eDNA as well) was dependent upon the T6SS of *B. cenocepacia*.

Given the affect that a T6SS mutant had on the relative amount of IHF and eDNA present in a biofilm built *in vitro* (Figure **S1**), we wondered whether IHF might possibly act *intracellularly* in *B. cenocepacia* to affect transcription of the T6SS gene cluster (*BCAL0340* to *BCAL0348*; the *BcsK* gene is equivalent to *BCAL0342*). As a first step in this regard, we examined the upstream region of this gene cluster and identified the sequence TCTCAACGATTTA, a near perfect match to the IHF binding consensus sequence WATCAANNNNTTR (where W is A or T, N is any nucleotide and R is a A or G). In the presence of 50 nM *E. coli* IHF a strong DNase footprint is visible (Figure **7** **, Panel D**) covering the region 25 to 52 bp upstream of the coding sequence of BCAL0340 and overlapping this match to the consensus sequence. This result could indicate that IHF self regulates its own release as well as perhaps that of eDNA that is incorporated into the biofilm matrix.

## Discussion

Cystic Fibrosis (CF) is a hallmark example of a chronic and persistent disease that defies current treatment modalities. Biofilms resident within the lungs of CF patients contribute significantly to both pathogenesis and chronicity. A biofilm is a highly-organized, multicellular community encased in an extra-cellular polymeric matrix or substance (or EPS) that is affixed to an inert or biological surface and is the preferred lifestyle of all bacteria in nature. Bacterial populations within a biofilm, as opposed to their planktonic or free-living counterparts, have a reduced growth rate (due to nutrient limitation), a distinct transcriptome [40,41] and a substantially increased resistance not only to effectors of innate and acquired immunity, but also to the action of antibiotics [42]. Moreover, the EPS presents a formidable physical barrier to phagocytic cells and other bacterial clearance mechanisms (both physical and physiological) and thereby, biofilms are highly recalcitrant to eradication [43]. Diseases wherein biofilms play a major role in pathogenesis and chronicity, such as CF, thus require novel methods for treatment and prevention. Whereas the composition of the EPS of biofilms is highly diverse among *genera*, as well as influenced by the environment in which it is formed, a very common and critical component is the incorporation of extracellular DNA (eDNA). Although the mechanism by which eDNA is released by the microbe and/or incorporated into the biofilm matrix has not yet been elucidated for many pathogens, eDNA is nonetheless of tremendous interest both in terms of its biological functionality, as well as its role as a structural component of the biofilm.

We’ve shown here that *Burkholderia cenocepacia* incorporates an abundance of eDNA into the biofilms it forms. The presence of this wealth of eDNA is likely to provide exceptional protection to resident *B. cenocepacia* as a physical barrier and, as we have recently shown, eDNA within a biofilm can also bind effectors of innate immunity [44], thus limiting or preventing their access to bacterial cells within. Specifically with regard to *B. cenocepacia*, Peeters et al. [45] showed that sessile *B. cenocepacia* were highly resistant to chlorhexidine, hydrogen peroxide and 5% bleach, even after treatment for 5 mins. Moreover, analysis of FDA product recall data for non-sterile pharmaceutical products from 1998–2006 showed that 48% of recalls were due to contamination by either *B. cepacia*, *Pseudomonas* species or *Ralstonia* *picketti* [46]. For non-sterile and sterile products, *B. cenocepacia* was the most frequently isolated species. Collectively, these data indicate that *B. cenocepacia* contamination of surfaces, equipment and pharmaceutical devices, likely in the form of a biofilm, serves as a source of infection for not only CF patients, but for any hospitalized, ventilated [14,47] and/or immunocompromised patient *without* CF [48]. These observations inspired us to attempt to develop a novel immunotherapeutic strategy for CF patients, particularly those infected with *B. cenocepacia*. To do so, we focused our attention on eDNA and a family of proteins known to bind to this extracellular DNA, the DNABII proteins.

What we’ve found to date is that *B. cenocepacia* incorporates an abundance of eDNA into their biofilms. This eDNA is associated with a DNABII protein that interacts with antiserum directed against isolated native IHF produced by *E. coli*. When *B. cenocepacia* biofilms are examined to determine the spatial distribution of this DNABII protein, we found that as observed for NTHI [20], positive labeling was associated with each vertice of crossed strands of eDNA present within the biofilm. Biofilms produced by *B. cenocepacia* were susceptible to disruption by anti-IHF but not naive serum in an *in vitro* assay system. Further, this disruptive effect of antiserum directed against IHF rendered bacteria within a *B. cenocepacia*-induced biofilm susceptible to the killing action of several antibiotics traditionally, but usually ineffectively, used to treat CF patients. These antibiotics were either not effective or were significantly less effective at killing *B. cenocepacia* within a biofilm *in vitro* in the absence of treatment with anti-IHF serum. Whereas the action of anti-IHF serum resulted in disruption of *B. cenocepacia* formed biofilms by targeting a lynchpin protein responsible for bending and stabilizing eDNA into the lattice structure seen within these biofilms, DNase was not found to be effective when incubated with *B. cenocepacia* biofilms *in vitro*. In fact, unlike the reductive effect observed with biofilms formed by either NTHI [22] or *P. aeruginosa* [49], treatment of a *B. cenocepacia* biofilm with DNase induced the formation of a markedly more robust biofilm, suggesting that treatment of CF patients who are infected with *B. cenocepacia* would be contraindicated. We also demonstrated that pre-treatment of *B. cenocepacia* with antiserum directed against IHF appeared to facilitated the routing of ingested *B. cenocepacia* to a more effective degradative pathway within murine CF macrophages as there was a statistically significant increase in killing of ingested *B. cenocepacia* that were treated with anti-IHF at 6 hours post-infection than were those that had been pre-incubated with naive serum. Whereas the mechanism(s) for this observation are not yet known, we hypothesize that binding of anti-IHF to bacterial cell-associated extracellular IHF may have played a role in this regard. Lastly, in an attempt to begin to unravel the molecular mechanism by which *B. cenocepacia* incorporates both eDNA and DNABII protein(s) into its biofilm, we examined biofilms built by both a T3SS and T6SS mutant of the strain K56-2 isolate used here. Whereas both mutant biofilms were compromised overall in terms of relative biofilm robustness, that built by the T6SS mutant was absent of labeling via antiserum directed against IHF, suggesting that secretion of this protein as well as robust biofilm formation was dependent upon an active T6SS. Moreover, we identified a putative IHF binding site directly upstream of the T6SS gene cluster, suggesting that IHF may regulate its own export. Indeed a formal transcriptomal analysis of IHF deficient mutants may further delineate IHF’s role in *B. cenocepacia* pathogenesis; it is already known that IHF is both part of the extracellular matrix and regulates virulence factor expression in uropathogenic *E. coli* (Justice, et al 2012). The mechanisms that underlie these observations are being further investigated.

Despite tremendous recent advances in our ability to better manage patients with CF, these individuals are only expected to survive until their mid-30s, even today. At least 90% of CF patients die of respiratory failure after enduring many years of chronic, recurrent and persistent bacterial infection of the lungs. Bacteria dwelling within a biofilm in the lungs of CF patients present a formidable obstacle. Thereby, in order to design novel and effective strategies to better treat and/or prevent the long-term bacterial infections characteristic of CF, it is necessary to understand both the unique biology of biofilms, as well as determine how one might undermine these structures to mediate a therapeutic or preventative ‘cure’. Herein, we showed that targeting a bacterial protein that stabilizes eDNA present within a biofilm is highly effective for reducing or eradicating that structure *in vitro*. Moreover treatment of a *B. cenocepacia* induced biofilm with anti-IHF worked synergistically with multiple standard antibiotics to render resident bacterial cells sensitive to killing. Lastly, we found that pre-treatment of *B. cenocepacia* with antiserum directed against IHF significantly inhibited their survival when ingested by murine CF macrophages. Based on data obtained to date, an approach which targets DNABII proteins associated with eDNA within a *B. cenocepacia* biofilm shows promise in providing a potential new approach for treatment of CF patients, and particularly those colonized with *B. cenocepacia*. Most importantly, given that multiple human pathogens appear to use a similar strategy wherein eDNA within the biofilm is associated with a member of the DNABII family of nucleic acid binding proteins, the approach developed here is likely to have utility for other respiratory tract pathogens which often precede and facilitate *B. cenocepacia* infection of the CF lung [2].

## Supporting Information

Figure S1 Demonstration of relative eDNA content of biofilms formed in chamber slides by either the parental isolate K56-2, its T3SS mutant or its T6SS mutant. Biofilms formed by either the parental *B. cenocepacia* strain or its T3SS and T6SS mutants followed by staining with FilmTracer FM 1-43 can be seen in Panels A,C&E, respectively (pseudocolored red). Relative eDNA content of each unfixed biofilm can be ascertained via immunolabeling of each biofilm with a monoclonal antibody directed against dsDNA as in Panels B, D &F (pseudocolored white).
