# Supplementary figures and images for: Structural Stability of Burkholderia cenocepacia Biofilms Is Reliant on eDNA Structure and Presence of a Bacterial Nucleic Acid Binding Protein
Source: PLoS One. 2013 Jun 14;8(6):e67629. doi: 10.1371/journal.pone.0067629 (PMC3682984; doi:10.1371/journal.pone.0067629)

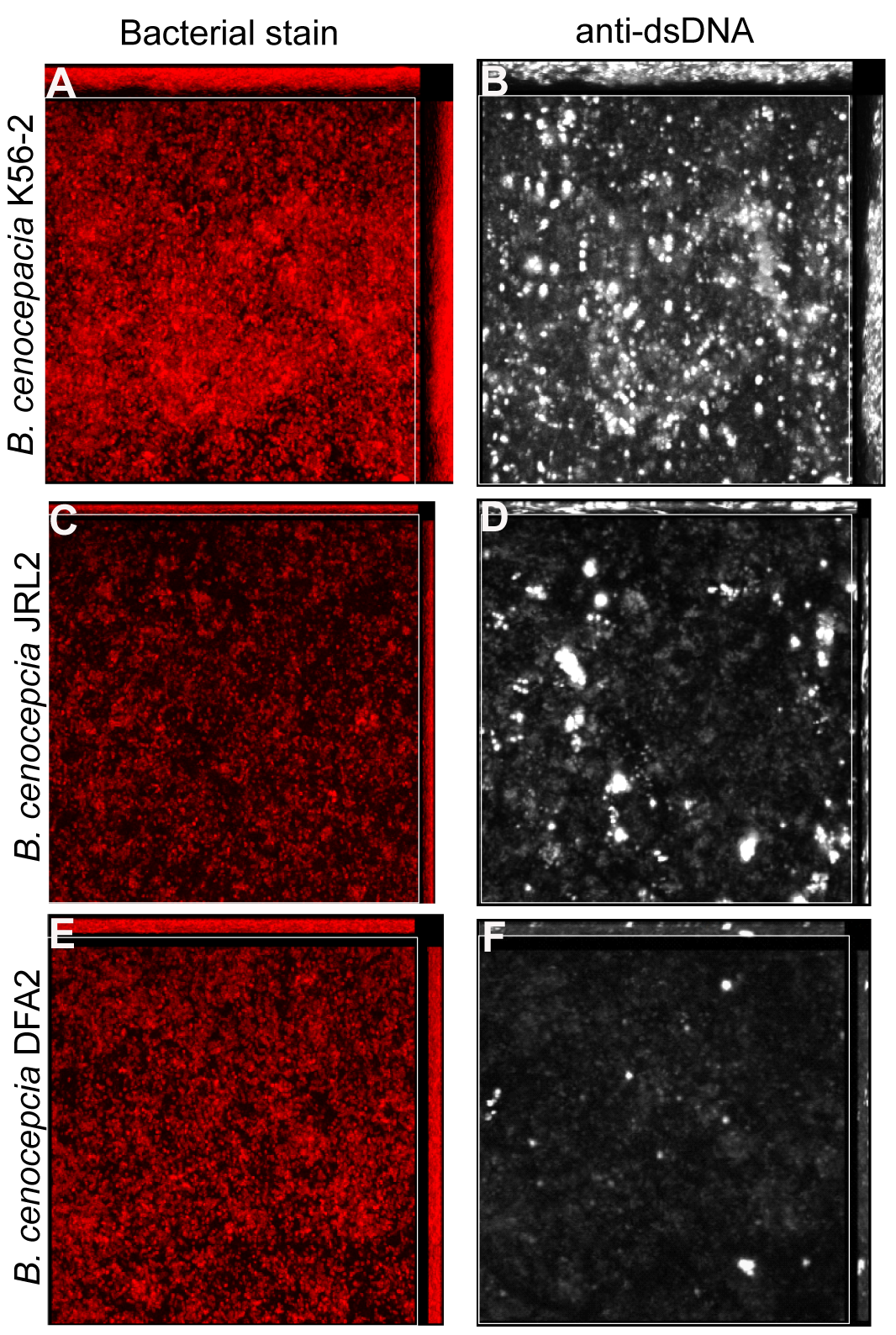

Supplement: Figure S1 — Biofilms formed by either the parental B. cenocepacia strain or its T3SS and T6SS mutants followed by staining with FilmTracer FM 1-43 can be seen in Panels A,C&E, respectively (pseudocolored red). Relative eDNA content of each unfixed biofilm can be ascertained via immunolabeling of each biofilm with a monoclonal antibody directed against dsDNA as in Panels B, D &F (pseudocolored white). [file pone.0067629.s001.tif]
